# A qualitative exploration of an autism-specific self-compassion program: The ASPAA

**DOI:** 10.1177/13623613241234097

**Published:** 2024-02-21

**Authors:** Chris Edwards, Vicki Gibbs, Abigail M A Love, Lydia Brown, Ru Ying Cai

**Affiliations:** 1 Autism Spectrum Australia, Australia; 2 Griffith University, Australia; 3 University of Sydney, Australia; 4 University of Melbourne, Australia; 5 North Eastern Rehabilitation Centre, Australia; 6 La Trobe University, Bundoora, Australia

**Keywords:** autistic adults, intervention, online, qualitative research, self-compassion

## Abstract

As mental health concerns coupled with inadequate supports have been described as reaching crisis proportions in autistic people, there is a pressing need for accessible and effective support systems. While self-compassion has shown various benefits in the general population, its application in supporting autistic individuals remains limited. This study investigated the experiences of 39 autistic adults who participated in an autism-specific online self-compassion program. The program included a series of five modules which incorporated psychoeducation, meditation, and self-reflective exercises that were completed over a 5-week period. Qualitative data was collected through weekly check-ins and a post-program survey, which underwent thematic analysis through the lens of an autistic researcher resulting in four key themes: the positive impact of self-compassion, challenges faced during the program, recognizing self-compassion as a journey, and the value of program adaptations. These findings shed light on the experiences of autistic adults engaging with self-compassion interventions, highlighting both the benefits and barriers they encountered. The study underscores the importance of developing tailored interventions that consider the unique needs and perspectives of autistic individuals to promote improved mental health outcomes and foster inclusivity.

**Lay abstract:**

Autistic people often struggle to find the right support for their mental health. We wanted to change that by trying a new approach to help autistic adults with their emotions and well-being. We focused on something called “self-compassion,” which is a way of being kind and understanding toward ourselves. This approach has worked well for many people, but we didn’t know if it would work for autistic individuals. We invited 39 autistic adults to join an online program that taught them about self-compassion. The program lasted 5 weeks and included educational materials, meditation exercises, and self-reflection activities. We asked the participants for feedback each week and at the end of the program. From their responses, we discovered four important things. First, self-compassion had a big positive impact on the well-being of autistic adults. Second, they faced some challenges during the program. Third, they saw self-compassion as a journey that takes time and practice. Finally, they described how they valued changes to help autistic people engage with the program. Our findings show that self-compassion can really help autistic adults. We learned about the benefits they experienced and the difficulties they faced. Most importantly, we found that personalized support is crucial for autistic individuals. By creating programs that consider their specific needs, we can improve their mental health and make their lives better.

Mental health concerns among autistic people, coupled with inadequate support, have been described as reaching crisis proportions (Mandy, 2022). This crisis is driven by higher rates of co-occurring conditions such as anxiety and depression, limited access to effective mental health treatments, and a lack of preparedness among healthcare professionals to assist autistic individuals with their health and well-being (Adams & Young, 2020; Camm-Crosbie et al., 2019; Corden et al., 2021; Hollocks et al., 2019; Lai et al., 2019; Maddox & Gaus, 2019). Autistic individuals are at a higher risk for loneliness (Grace et al., 2022), sleep difficulties (Richdale & Baglin, 2015), suicidal ideation (Dell’Osso et al., 2019; Hedley et al., 2017), premature mortality (Hirvikoski et al., 2016), decreased employment opportunities (Scheeren et al., 2021), and reduced quality of life (Mason et al., 2019). Mental health supports such as cognitive behavioral therapy (CBT) and mindfulness-based therapy (MBT) can be effective for autistic people. However, they may not be effective or accessible to all autistic people (Benevides et al., 2020; Camm-Crosbie et al., 2019).

Rather than solely focusing on psychological vulnerabilities, cultivating self-compassion is an alternative approach to mental health care widely applied in the general population, yet its exploration within the autistic community remains limited, with calls for further research and development of evidence-informed programs (Cai & Brown, 2021; Cai et al., 2023; Galvin & Richards, 2023; Wilson et al., 2022). Self-compassion, defined as a healthy way of relating to oneself with kindness during moments of failure or suffering, comprises three interconnected elements: self-kindness, common humanity, and mindfulness (Neff, 2003, 2023). Extensive research across different populations (e.g. people with psychotic disorders, trauma, binge eating, and anxiety) has found relationships between self-compassion and numerous mental health and well-being benefits, including increased life satisfaction, mindfulness, happiness, resilience, and improved sleep quality, as well as decreased stress, depression, and anxiety (Brown et al., 2021; Cleare et al., 2019; Ferrari et al., 2019; MacBeth & Gumley, 2012; Zessin et al., 2015). Furthermore, self-compassion has been recognized as a trainable skill that can be enhanced over time and through practice (Ferrari et al., 2019). However, there is a scarcity of research examining self-compassion within the autistic community.

Preliminary studies indicate that autistic adults self-report lower levels of self-compassion than their non-autistic counterparts (Cai et al., 2022; Galvin & Richards, 2023). As part of exploring why this may be the case, participants who shared their self-compassion experiences revealed tendencies toward self-criticism, emotional over-identification, and a sense of disconnection from others (Cai et al., 2022). These characteristics contrast with Neff’s (2003) three necessary elements of self-compassion: self-kindness, mindfulness, and common humanity. This aligns with autism research in related fields as to why autistic people may experience lower levels of self-compassion, such as internalizing stigma (Han et al., 2021) and heightened rates of loneliness (Grace et al., 2022) and victimization (Trundle et al., 2022). Furthermore, Cai et al. (2023) found that self-compassion influences mental health among autistic adults through emotion regulation, which is particularly relevant given the challenges autistic individuals face in emotional regulation and the associated impact on mental and physical health (Cai et al., 2018; Cibralic et al., 2019; Conner et al., 2020; White et al., 2014). These findings underscore the potential benefits of self-compassion practices for autistic adults, highlighting the importance of developing approaches to help them cultivate self-compassion skills.

In the general population, research has shown that online programs can serve as a viable method for promoting mental health in various areas, including depression, anxiety, well-being, stress, and mindfulness. Such programs have been demonstrated to provide benefits that are comparable to face-to-face services (Bégin et al., 2022; Saddichha et al., 2014; Sommers-Spijkerman et al., 2021; Spijkerman et al., 2016). Similarly, online self-compassion programs have demonstrated benefits, with notable increases in self-compassion, life satisfaction, and happiness and decrease in self-hatred, anxiety, stress, depression, and emotional regulation difficulties (e.g. Beshai et al., 2020; Finlay-Jones et al., 2017; Krieger et al., 2016; Nadeau et al., 2020). Notably, these gains have remained stable over time (Finlay-Jones et al., 2017; Krieger et al., 2016). Other advantages to online programs for users include being affordable, completable in private, self-paced, repeatable, and different modalities to support learning styles (Bégin et al., 2022).

Despite the potential benefits of online mental health programs, little research has explored their utilization among autistic individuals (Gaigg et al., 2020; Lunsky et al., 2022; Redquest et al., 2022), particularly concerning self-guided self-compassion concepts (Cai et al., this issue). Initial investigations indicate the potential of virtual mindfulness programs for autistic adults, though existing programs have predominantly relied on sessions with facilitators (Lunsky et al., 2022; Redquest et al., 2022). Gaigg et al. (2020) found promising results with online self-guided CBT and MBT tools in reducing anxiety among autistic adults. They highlighted the need for researchers to develop autism-specific online mental health tools that build upon existing research to ensure efficacy and accessibility. Despite emerging evidence supporting the effectiveness of self-guided online programs in promoting the mental health of autistic individuals, no studies to date have examined this delivery method within the context of self-compassion.

To address this gap, we co-produced a self-guided program (Aspect Self-compassion Program for Autistic Adults; ASPAA) grounded in evidence-based interventions such as the Mindful Self-Compassion program (Germer & Neff, 2019; Neff & Germer, 2018) and Compassion-Focused Therapy (Gilbert, 2014). The content was adapted for an autistic audience with an autistic researcher (C.E.) and an autistic advisory group. We have evaluated the feasibility and acceptability of the program and found it to be both feasible and acceptable (Cai et al., this issue). In addition, we found that autistic adults who completed the ASPAA experienced increased levels of self-compassion and positive emotions, alongside decreased symptoms of anxiety, depression, and emotion regulation difficulties from pre- to post-program.

However, mental health programs can cause harm or unintended consequences, factors that should be reported but are often overlooked (Papaioannou et al., 2021). Self-compassion practices, in particular, can be distressing or triggering for people who are psychologically vulnerable as it includes reflecting on emotional experiences (Germer & Neff, 2015), a factor particularly relevant for the autistic community given the rates of victimization (Trundle et al., 2022). Even though positives have been reported for the ASPAA pre- and post-program, qualitative methods are required as part of a comprehensive evaluation to gain insight into participant perspectives (Pellicano et al., 2014). While qualitative methods can capture any unintended harms, it can also provide insight into how the ASPAA led to quantifiable improvements and areas for improvement in the program. Therefore, this study aimed to qualitatively capture the experiences of autistic adults who completed the ASPAA.

## Method

### The Aspect Self-compassion Program for Autistic Adults

The ASPAA is a five-module course including psychoeducation, meditation, and self-reflective exercises that draw from evidence-based self-compassion-based interventions, including the Mindful Self-Compassion program (Germer & Neff, 2019; Neff & Germer, 2018) and Compassion-Focused Therapy (Gilbert, 2014). Its development involved the contribution of both autistic and non-autistic researchers. During the initial phase of program creation, individual interviews were conducted with members of an autistic advisory group (totaling three individuals) to ensure the content, wording, and exercises were adapted to suit an autistic audience. Members of the advisory group were selected as they represented “low,” “moderate,” and “high” levels of self-compassion and were reimbursed for their time. Upon completing the program modules, the advisory group collectively reviewed all modules and provided feedback during a 2-h focus group. Based on this feedback, the content and structure of the ASPAA underwent further refinement before the commencement of the current study. Each advisor also recorded a short video discussing their self-compassion and self-critical experiences.

The content was created in PowerPoint and imported into Articulate 360, where the modules were exported as web pages. These were added to Canvas (https://canvas.instructure.com/), a learning management platform, where additional interactive/accessible features were included, as well as controlled access. It was intended for participants to access Canvas across a range of devices; however, due to the limitations of Canvas, the program was not accessible on tablets or phones, only desktops and laptops.

A summary of the ASPAA, including the exercises and skills, is shown in Table 1, detailing what was included over the 5 weeks and five modules. Before commencing, participants completed a short introductory module (Module 0) which provided background information and expectations for the program. Participants were encouraged to stop exercises/skills if they brought out strong negative emotions and to seek additional support or contact C.E. or R.Y.C. This included a list of support organizations (e.g. Beyond Blue & Lifeline) being provided to all participants and a clinical psychologist (V.G.) being available to provide referrals for additional support if needed.

**Table 1.** The Aspect Self-compassion Program for Autistic Adults (ASPAA).

Module	Description	Exercise^a^	Daily skill^b^
0: Introductory module	This module provided background information about what to expect in the ASPAA (e.g. purpose, structure, where to do it, how often, helpful tips, and how to ask questions)	N/A	N/A
1: Understanding self-compassion	An introduction to the three elements of self-compassion, and describing what self-compassion is not	How do I treat a friend or loved one?: A written exercise that encouraged participants to recognize how they may treat friends/loved ones better than themselves	Soles of your feet: An audio recording that guided participants through a grounding exercise that involved focusing on the soles of the feet
2: Benefits of self-compassion	How research suggests that self-compassion can benefit autistic adults in areas such as anxiety, depression, and well-being	Community humanity: A written exercise where participants were asked to read through common challenges experienced by autistic adults and reflect that these shared experiences can be a way of connection	Soothing touch: A written exercise where participants were provided with internal and external examples of soothing touch, with the goal of finding a form of touch that felt safe and comfortable
3: Mindfulness	Introducing the mindfulness component of self-compassion and providing examples for how it can be practiced daily	5-4-3-2-1 method: A YouTube video that guided participants through their five senses as a calming technique	Progressive muscle relaxation: An audio recording that guided participants through an exercise of tensing and relaxing muscles in the body
4: Finding your compassionate voice	Supporting participants to become kinder toward themselves, particularly through internal dialogue	Loving-kindness phrases: A written exercise designed to help participants create loving-kindness phrases that were deeply meaningful to them	Loving-kindness meditation: A short YouTube meditation video that was guided by the loving-kindness phrases
5: Accepting our experiences	Supporting participants to become more accepting of self-compassion and less resistant to unpleasant experiences	Reflection about resistance: A written exercise that helped participants become more aware of resistance in their lives	Three self-compassion gestures: A YouTube video that included three gestures that reflected the three self-compassion elements

a Generally one-off exercises although they could be completed multiple times.

b Participants were asked to complete these skills on a daily basis throughout the program, only practicing the skill for that week. During Module 3: Mindfulness, participants were provided the choice of either 5-4-3-2-1 or progressive muscle relaxation as their “daily skill” for that week, selecting the option that was most comfortable.

### Participants

Thirty-nine autistic adults participated in the research and completed the ASPAA (28 female, 5 male, 6 non-binary or agender; *M*_age_ = 45.28, *SD*_age_ = 11.92). Most participants (*n* = 30) reported a formal autism diagnosis and provided evidence for a researcher to confirm. The remaining participants (*n* = 9) self-identified as being autistic. A summary of participant demographics is shown in Table 2.

**Table 2.** Participant demographic information (*N* = 39).

	*n* (%)
Gender	
Male	5 (13)
Female	28 (72)
Non-binary	5 (13)
Agender	1 (3)
Autism status	
Formal diagnosis	30 (77)
Self-identify	9 (23)
Ethnicity	
White	38 (97)
Mixed	1 (3)
Co-occurring conditions	
Anxiety	28 (72)
Depression	26 (67)
Speech or language impairment	2 (5)
ADHD	12 (31)
OCD	4 (10)
Sensory processing disorder	4 (10)
Seizure disorder (epilepsy)	2 (5)
Other	12 (31)
No co-occurring conditions	3 (8)
Highest level of education	
School certificate	1 (3)
High school certificate	3 (8)
TAFE or diploma	2 (5)
Undergraduate studies at university	13 (33)
Postgraduate studies at university	20 (51)
Current employment status	
I am not working	12 (31)
I am working casually	5 (13)
I am working part-time	12 (31)
I am working full-time	10 (26)
Current education status	
I am not studying	30 (77)
I am studying part-time	7 (18)
I am studying full-time	2 (5)
Current relationship status	
Single	18 (46)
Married	16 (41)
In a de facto relationship	4 (10)
Rather not say	1 (3)

ADHD: attention-deficit/hyperactivity disorder; OCD: obsessive-compulsive disorder; TAFE, technical and further education.

### Procedure

The ethical components of this research were approved by the University of Sydney Human Research Ethics Committee (Project No.: 2022/598). Participants were recruited through Autism Spectrum Australia’s (Aspect’s) social media and mailing lists, autism support networks, and word of mouth. As previous researchers have recommended that caution be exercised when introducing self-compassion practices to autistic people (Cai & Brown, 2021), potential participants were screened through a suitability survey. To ensure the safety and well-being of participants who may be psychologically vulnerable and prone to being severely triggered by self-compassion exercises, we established exclusion criteria:

- Presence of a current or lifetime psychotic disorder;
- Recent suicide ideation or suicidal behaviors;
- Recent initiation or cessation of psychiatric medication or psychotherapy.

As the ASPAA was created in collaboration with autistic adults without an intellectual disability, with the purpose of developing a self-guided program to cultivate self-compassion, participants were included if they satisfied the following criteria:

- They were 18 years of age or older;
- They provided evidence of a formal autism diagnosis, or they self-identified as autistic and scored above the Autism Spectrum Quotient-Short (AQ-Short; Hoekstra et al., 2010) cut-off of 65;
- They did not have an intellectual disability;
- They scored “low” to “moderate” on the Self-Compassion Scale (SCS; Neff, 2003; range: 1 to 3.5);
- They resided in Australia;
- They reported having an Internet connection with a computer or laptop that could listen to audio.

Participants suitable for the ASPAA were directed to complete a pre-program survey that collected demographic information and included various mental health measures (see Cai et al., this issue, for more information).

After each module, participants engaged in a weekly check-in with C.E. or R.Y.C. depending on their communication preference (email, phone, or Zoom). The primary objective of this check-in aimed to provide participant support in case of any potential discomfort arising from completing the module. Check-ins were completed by the two lead designers/researchers of the ASPAA, C.E. and R.Y.C. C.E. is autistic, whereas R.Y.C. is non-autistic and experienced as a self-compassion practitioner. Each participant was paired with one of the two lead researchers for their weekly check-in throughout the program. Thirteen of the 39 participants preferred email and provided their answers directly to their paired researcher. This often included a back-and-forth email conversation between participant and researcher to debrief as needed. The remaining 26 participants preferred a phone or Zoom weekly check-in. These check-ins were typically 10–20 min long, with the researcher typing the participant’s responses verbatim, and these responses were then saved as a Microsoft Word file. Each check-in followed the same structure, where participants were asked questions such as how they experienced the prescribed exercises, how they practiced the daily skills, what they found most helpful, what they did not like, and how the module could be improved. These questions were attached to the end of each module so that participants could prepare for the check-in as needed.

Participants completed a post-program survey after completing Module 5 and the scheduled weekly check-in. This survey included repeated measures from the pre-program survey (see Cai et al., this issue, for more information) and several open-ended questions where participants were asked to reflect on their experiences throughout the ASPAA. Participants were asked questions about what they found most helpful, what they did not like, how the ASPAA could be better, how they thought self-compassion might help them, and whether they thought the ASPAA would help other autistic adults.

### Data analysis

Qualitative data analysis was guided by a six-phase reflexive thematic approach: data familiarization, initial coding, generating themes, reviewing and developing themes, refining the themes, and producing the report (Braun & Clarke, 2006, 2019). This process included C.E. first familiarizing with the qualitative data while merging each participant’s weekly check-in with their post-program survey into a CSV file for combined analysis. This file was imported into NVivo to commence initial coding. The coding was guided by an inductive approach, using a combination of semantic and latent coding through the lens of an autistic researcher. This provided valuable insight into the data as an “insider-researcher” (Greene, 2014), exploring the self-compassion experiences of autistic adults through the lens of an autistic adult beginning their self-compassion journey. The codes were continuously refined with the research question in mind, leading to the generation of initial themes. C.E. and R.Y.C. discussed the initial themes in relation to the research question, coded extracts, and the entire data set. The themes were discussed following a similar process with the wider research team. Further refinements were made before preparing the results.

### Community involvement statement

This research was conducted by the Aspect Research Centre for Autism Practice (ARCAP), a participatory research center that includes autistic and non-autistic researchers. This research was led by autistic (C.E.) and non-autistic (R.Y.C.) researchers as the lead designers of the ASPAA. The wider research team included a parent (V.G.) and a sibling (A.M.A.L.) of autistic adults and a self-compassion researcher/clinical psychologist (L.B.). Research team members have professional experience across education, psychology, and health. This research also included a team of three autistic advisors who informed the autism-specific adaptations and recorded videos for inclusion in the ASPAA.

## Results

The reflexive thematic analysis led to the generation of four themes that represented the experiences of autistic adults who completed the ASPAA: (1) “It’s helping me be easier on myself. More loving towards myself,” (2) Practicing self-compassion can be difficult and emotional, (3) “Journey of self-awareness that takes time and practice with a variety of tools,” and (4) Adaptations for online programs are valued. Each quote includes a participant identification number and gender identity, followed by the specific exercise/skill mentioned in some instances (these are described in Table 1).

### Theme 1: “It’s helping me be easier on myself. More loving towards myself.”

Autistic adults who participated in the ASPAA shared how practicing self-compassion was calming, challenged their current ways of thinking, brought a sense of connection, and permitted them to be kind to themselves. One participant summarized the benefits of practicing self-compassion: “Being kinder and more gentle in my thoughts, reduces stress and anxiety, promotes feeling more calm and relaxed and more able to function” (ID:2, Female). These calming benefits were directly related to the exercises and skills that participants were asked to practice over the 5 weeks, and the fact that “many people do not know these exercises so they talk negatively and it’s a snowball effect. These exercises can give them a calm mind and body. Hopefully lessen anxiety” (ID:25, Female). The variety of skills provided participants different ways to “self-soothe or ground myself to help disrupt all of the thoughts flying around in my head” (ID:12, Female). For example, practicing one particular skill helped a participant “get through yesterday . . .” (ID:36, Female, 5-4-3-2-1) and another “to feel calm. Because I used it as a tool during the week . . . It stopped a massive escalation” (ID:22, Male, 5-4-3-2-1). Another example of a skill “we will be putting into our toolbox” (ID:21, Female) was practicing “soothing touch” as it is calming “to connect with my body” (ID:25, Female) and it “grounds me” (ID:14, Female).

Learning about self-compassion also provided several insightful moments for participants in terms of their thinking styles. One of the most revealing exercises for participants was the “how do I treat a friend or loved one?” exercise in Module 1. This exercise “raised awareness” and was viewed by one participant as a necessary “first step for self-compassion” (ID:9, Female). It helped participants understand that they were “much harsher on myself than everybody else” (ID:23, Female) and that they “found out how horrible I treated myself” (ID:6, Female).

Explaining concepts such as backdraft (initial surge of difficult emotions when practicing self-compassion) and resistance (psychological barriers to embracing self-compassion) resonated with participants. “(Backdraft) it’s an explanation for something I experience. I struggle to define and feel things. Backdraft resonates . . . Explains a lot of my life . . . helped me understand and become honest with how I feel” (ID:31, Female). Similarly, “(resistance) it’s something that I haven’t heard discussed in this way before, and a topic that I’ve been trying to articulate properly to therapists for years but have felt they haven’t really ‘got’ what I meant” (ID:14, Female). Overall, many participants acknowledged that the information, reflections, and skills as part of the ASPAA helped them understand themselves more than before.

Developing self-compassion provided a sense of connection for participants in our study. This sense of connection was often shared after hearing the self-compassion experiences of the autistic advisors (Chris, Anna and Aiyah) that their experiences “helped to have that sense of community and connection with other autistic people” (ID:4, Non-Binary). For instance, Chris’ video “made me not feel so alone . . . it really helped to know that I’m not the only one that feels that way” (ID:6, Female), and both Anna’s and Aiyah’s videos “resonated with me” (ID:7, Non-Binary and ID:25, Female). Furthermore, the program’s focus on common humanity (a fundamental element of self-compassion) was described as helping the participants recognize “that pain or mistakes link us not separate us” (ID:13, Female) and that by “understanding that it’s (feeling bad) a common thing, or something felt by others is permission to feel that way” (ID:30, Female).

Finally, participants felt that the ASPAA helped give them “permission to be kind to self” (ID:13, Female). The knowledge around self-compassion that they gained helped them with “understanding that being kind to yourself is not selfish but healthy to help you gain skills to cope with life” (ID:13, Female). Some acknowledged that “being kinder to myself will help me better regulate unwanted feelings and bad days” (ID:14, Female). Notably, “by liking ourselves, we will be kinder to ourselves, more understanding of our being different, over time hopefully we will not be so mean to ourselves” (ID:21, Female).

### Theme 2: practicing self-compassion can be difficult and emotional

While participants detailed the skills and knowledge gained through engagement in the ASPAA, they also spoke of how difficult and emotional it was to become more self-compassionate. For some participants, the challenge was “maintaining focus and concentration” (ID:1, Male), particularly as their minds would often go “off on different tangents” (ID:12, Female) during exercises/skills. Another difficulty shared by participants included having “little insight into our feelings” (ID:11, Female), which made it difficult to complete exercises/skills that included emotional components. Not surprisingly, the effort required to maintain regular self-compassion practices was hard as it “takes a lot of mental energy” (ID:15, Male).

Parts of the ASPAA were inaccessible for some participants as the exercises/skills were inappropriate for their sensory profile. For example, one participant stated that they could not complete an exercise that asked them to focus their attention on the soles of their feet, saying they could not “continue with the exercise as the sensation of my feet was too weird” (ID:13, Female). Another participant found the progressive muscle relaxation exercise challenging, reporting that they could not “concentrate on my physical body—that’s a sensory trigger. At 10 mins I had to stop and stim/rock to regulate” (ID:32, Female). Some participants also disliked some of the more cognitive exercises that included sensory components such as the “5-4-3-2-1” skill, “these things are not good or suitable for me” (ID:2, Female).

Some participants shared that developing their self-compassion skills was emotional and often uncomfortable. Some exercises/skills were described as being “painful” (ID:7, Non-Binary, Loving-kindness meditation), “confronting . . . it was rather horrible” (ID:23, Female, Common humanity), and “quite uncomfortable” (ID:4, Non-Binary, Soles of your feet). Exercises that prompted deeper reflection were even more emotional, including reflections such as “it brought up painful memories” and “I started crying and kept crying for the next few hours” (ID:7, Non-Binary, How do I treat a friend or loved one? and Loving-kindness phrases). While lived experience videos were valued, they were the source of distress in one particular instance: a participant compared their progress to that in the video, “I ended up beating myself. Comparing myself to other people. Criticizing myself” (ID:15, Male). As some participants shared emotional experiences in practicing self-compassion, they completed exercises/skills “with the help of our support worker” (ID:21, Female), or it was recommended that it should be a “collaborative process with a psychologist” (ID:20, Female) or completed under the “guidance of a therapist or counselor” (ID:35, Female) as some parts can be dysregulating.

### Theme 3: “Journey of self-awareness that takes time and practice with a variety of tools”

Autistic adults in our sample described how developing their self-compassion skills was “hard at first, but it is becoming easier” (ID:26, Non-Binary) and that it is “worth persevering” (ID:11, Female). Participants acknowledged that the journey to self-compassion involves “a slow learning curve. However, I can tell that it will make a difference” (ID:33, Female). Participants described how difficult becoming self-compassionate was and that through time and practice, “I think it will get easier” (ID:23, Female). This hope for the future was shared by other participants, such as one person who “started to feel a spark, but it went away. Maybe the more I do it, the more I can focus on it and feel love for myself” (ID:8, Non-Binary).

While participants expressed initial discomfort and difficulty, many also acknowledged that “the initial discomfort faded” (ID:4, Non-Binary) over time, such as how “Loving-kindness phrases” “became less uncomfortable to hear” (ID:29, Male). In fact, through practice, being self-compassionate was becoming “slightly more instinctual” (ID:4, Non-Binary) and “more natural and more enjoyable” (ID:9, Female). When compared to the start of their journey, one participant acknowledged “that I’ve actually come a long way . . . I’m not as harsh on myself as I used to be” (ID:10, Non-Binary), and another felt that some skills have even become “essential for my daily ability to cope with stressors” (ID:4, Non-Binary).

### Theme 4: adaptations for online programs are valued

Autistic adults in our sample shared how they valued adaptations to support an autistic audience. Notably, they expressed it was “great to hear autistic voices” (ID:1, Male), referring to videos of autistic people that were included in the ASPAA and that they wanted “more videos with autistic people” (ID:18, Female) to help connect the content with their own experiences. As some exercises were challenging, participants “found the examples most useful” (ID:5, Female) in guiding them but also wanted “a little bit more prompt/support” (ID:20, Female) to help reinforce that they were on the right track. Participants expressed more consideration to the language used in the program, such as “simpler language, but not in a patronizing way” (ID:8, Non-Binary) or rather than “take a minute—(it) didn’t take a minute. Use take a moment” (ID:16, Female) instead. Furthermore, multiple participants recommended the inclusion of references and external links, “would be helpful to have links to original source” (ID:16, Female) as “I’m always up for more information, additional reading/resource at the end of each module” (ID:9, Female).

Many participants emphasized the importance of accessibility for an online program to support autistic adults. This included flexibility in the exercise/skill to practice depending on their preferences “so that if one doesn’t suit, then another can be used” (ID:13, Female). Participants expressed they did not like some exercises/skills “and the way they were prescriptive” and preferred “freedom and guidance” in finding what “worked for us authentically” (ID:15, Male). While some recommended modifications came from the researchers, other participants modified exercises to suit their needs, “I will still try the activity, but with adjustments to allow for my own engagement” (ID:3, Female). Similarly, participants valued the presentation of content that catered to “different learning styles” (ID:23, Female), such as videos with transcripts “as my auditory processing isn’t great” (ID:14, Female). However, some participants desired enhanced accessibility, requesting “more visuals/different ways of presenting content that isn’t words . . . alternatives to reading” (ID:9, Female). They also emphasized the importance of having resources to help facilitate skill practice, such as “a visual sheet/guide . . . that could be downloaded by participants and kept as a reference to come back to” (ID:4, Non-Binary).

## Discussion

The present qualitative investigation sought to capture the experiences of autistic adults who participated in an autism-specific online self-compassion program, the ASPAA. Although the ASPAA is feasible and acceptable for autistic adults, and positive outcomes have been reported in domains of self-compassion, anxiety, depression, and emotion regulation (Cai et al., this issue), it is also essential to understand how the autistic participants perceived various aspects of the program and whether there are opportunities for improvement as part of a comprehensive evaluation. This investigation complements the quantitative findings in our current work (Cai et al., this issue), addressing a direct response to calls made by researchers for evidence-based self-compassion programs to promote the well-being of autistic adults (Cai & Brown, 2021; Cai et al., 2023; Galvin & Richards, 2023; Wilson et al., 2022) while also considering any harms or unintended consequences (Papaioannou et al., 2021).

Participants described how they experienced benefits and difficulties in practicing self-compassion but recognized that it takes time and practice to become more self-compassionate. Participants reported that they developed a more accepting and kinder attitude toward themselves, better regulation of challenging emotions, and an increased sense of connection with others. These experiences are consistent with Neff’s (2003) conceptualization of self-compassion and provide support for the notion that self-compassion can be developed and can lead to substantial improvements in the lives of autistic adults (Cai et al., 2023; Ferrari et al., 2019). The findings suggest that fostering self-compassion may be a feasible strategy for addressing internalized stigma and managing emotion regulation difficulties in autistic individuals (Botha et al., 2020; Cai et al., 2018; Conner et al., 2020; Han et al., 2021; White et al., 2014). Furthermore, developing an enhanced sense of connection (common humanity element) is of critical significance given the association between belonging and well-being and the existing knowledge on loneliness in the autistic community (Camm-Crosbie et al., 2019; Milton & Sims, 2016; Grace et al., 2022).

Although there was positive feedback overall about the benefits of self-compassion, participants also spoke of challenges when practicing self-compassion throughout the ASPAA. Some of these challenges related to known areas of differences in the autistic community, such as executive functioning (e.g. working memory) (Davis et al., 2021; Desaunay et al., 2020), instructional methods that were not appropriate (Davis et al., 2021), and varying sensory profiles (Sibeoni et al., 2022). We attempted to mitigate risk by excluding participants who were deemed psychologically vulnerable and conducting weekly check-ins (Cai & Brown, 2021; Gaiswinkler et al., 2019; Germer & Neff, 2015). Nevertheless, some participants experienced difficulty and/or emotional distress. These instances underscore the significance of incorporating multi-modal learning opportunities, offering alternative exercises tailored to individual needs, especially when sensory components are involved, and explicitly communicating the acceptability of practicing self-compassion in collaboration with a trusted person or a trained professional.

We also identified ways ASPAA could be improved to better support autistic adults. While we incorporated adaptations for an autistic audience as recommended by Gaigg and colleagues (2020), our findings show how vital these autism-specific adaptations are, and that seeking feedback from participants about specific programs is needed as additional adaptations were identified. For instance, our participants wanted more autism-specific content and lived experience videos. Further revisions related to broader “autism-friendly” principles that have been well documented in other areas, principles such as flexibility and adaptability (Jones, 2022), accessible language (Haydon et al., 2021; Sarrett, 2017), references for additional reading, multi-modal learning opportunities (Clouder et al., 2020; Davis et al., 2021), and the option to engage on a tablet or mobile (Bégin et al., 2022).

### Recommendations for research and clinical practice

The presence of an autism-related mental health crisis has been extensively documented in the literature (Adams & Young, 2020; Camm-Crosbie et al., 2019; Corden et al., 2021; Hollocks et al., 2019; Lai et al., 2019; Maddox & Gaus, 2019; Mandy, 2022), underscoring the importance of creating compelling and accessible mental health interventions for autistic adults. This qualitative exploration of participant experiences combined with our pre- and post-measure evaluation (Cai et al., this issue) supports the ASPAA, and self-compassion, as a worthwhile avenue for further exploration. However, as an emerging area, further research is needed, particularly with larger and more diverse samples (e.g. varying support needs and ages). Moreover, there is a requirement for research designs that more comprehensively assess the efficacy of a program like the ASPAA, incorporating methodologies such as randomized controlled trials and the systematic integration of follow-up data collection.

Our findings also provide some insight into ways in which future online mental health programs for autistic adults can be adapted, including:

- An autism-specific program should include autistic voices and autism-specific videos where possible rather than generic content.
- When providing exercises or skills to practice, ensure there are alternative options and make it clear that personalization is possible, particularly considering people’s sensory needs.
- Autistic adults appreciate having examples and prompts to help them gauge their progress.
- Ensure the language is “autism-friendly” and maintain a clear program structure.
- Include optional reading materials for those interested in further exploring the topic.
- Present content in a way that caters to different learning styles (e.g. voiceover, text, visuals, interactions, transcripts).
- A platform that works across various devices would be helpful, particularly as a smartphone application.

### Strengths and limitations

The present study possesses a notable strength derived from its foundation in a substantial body of self-compassion research spanning more than two decades (Neff, 2003, 2023; Neff & Germer, 2018). Moreover, the research process was enriched by a collaborative approach involving an autistic researcher and autistic advisors, thereby facilitating the adaptation of empirical evidence for an autistic population. By gathering data weekly and accommodating participants’ preferred communication modalities (e.g. Zoom, phone, or email), we could comprehensively explore individual experiences.

Several limitations should be acknowledged. The weekly check-ins with a researcher restricted participant recruitment to individuals residing in Australia. Furthermore, the study predominantly attracted participants identifying as female, White, and possessing university qualifications, thus potentially limiting the generalizability of the findings. The requirement for accessing the ASPAA as an online program imposed a barrier for autistic adults lacking access to technology, potentially excluding valuable participants who could have benefited from non-technological alternatives. Furthermore, we intended for the ASPAA to be accessible across various devices; however, due to technical challenges associated with Canvas, ASPAA did not work on tablets or phones. Finally, participant feedback indicated that the 5-week program duration might have been insufficient for adequate self-compassion learning and practice, with some expressing the potential benefits of a lengthier program.

## Conclusion

In conclusion, this study significantly contributes to the emerging research on self-compassion and autism by exploring an autism-specific online program previously not investigated. The positive results from the ASPAA’s pre- and post-measures demonstrate its effectiveness (Cai et al., this issue), while the perspectives of participating autistic adults emphasize the importance of considering their experiences in designing innovative approaches for mental health support. Incorporating participant feedback for continuous program refinement is crucial for ensuring efficacy and relevance. This approach acknowledges the value of the autistic community’s voice, leading to more effective interventions and improved mental health outcomes.

This study highlights the significance of a comprehensive approach to supporting the diverse needs of the autistic community. Programs like ASPAA address mental health challenges, creating a more inclusive and supportive environment. The findings contribute to knowledge on self-compassion and autism, paving the way for further research and evidence-based interventions prioritizing autistic individuals’ well-being. Ultimately, this study underscores the importance of considering the autistic experience in comprehensive evaluations, promoting mental health and inclusivity.
